# Regulation of the antibiotic elution profile from tricalcium phosphate bone cement by addition of bioactive glass

**DOI:** 10.1038/s41598-024-53319-2

**Published:** 2024-02-02

**Authors:** H. K. Abd El-Hamid, Mohammad M. Farag, Mohamed Abdelraof, R. L. Elwan

**Affiliations:** 1 Refractories, Ceramics and Building Materials Department, National Research Centre (NRC), El-Buhouth St., Dokki, 12622 Cairo Egypt; 2 Glass Research Department, National Research Centre (NRC), El-Buhouth St., Dokki, 12622 Cairo Egypt; 3 Microbial Chemistry Department, Biotechnology Research Institute, National Research Centre (NRC), El-Buhouth St., Dokki, 12622 Cairo Egypt

**Keywords:** Drug discovery, Materials science, Nanoscience and technology

## Abstract

This work aimed at tailoring of different properties of antibacterial drug delivery Ca-phosphate cements by incorporation of bioactive glass (BG). The cements were prepared from beta-tricalcium phosphate cement (β-TCP) and BG based on 50 SiO_2_—20 CaO—15 Na_2_O—7 B_2_O_3_—4 P_2_O_5_—4 Al_2_O_3_ wt% with different percentages of BG [5, 10, 15, and 20% (w/w)]. The composite cements were characterized by XRD, FTIR, and TEM. Moreover, in vitro bioactivity and biodegradation were evaluated in the simulated body fluid (SBF) at 37 °C. In addition, physical properties and mechanical strength were determined. Also, the effect of glass addition on the drug release profile was examined using gentamicin. Finally, the antimicrobial activity was studied against *Staphylococcus aureus*, *Pseudomonas aeruginosa*, and *Klebsiella pneumonia* bacteria, one unicellular fungal strain (*Candida albicans*), and one multicellular fungal strain (*Mucor racemosus*)*.* The results showed that after soaking in SBF, the compression strength values ranged from 14 to 36 MPa, the bulk densities and porosities were within 1.35 to 1.49 g/cm^3^ and 51.3 to 44.71%, respectively. Furthermore, gentamicin was released in a sustained manner, and BG decreased the released drug amount from ~ 80% (in pure β-TCP) to 47–53% in the composite cements. A drug release profile that is sustained by all samples was achieved. The antimicrobial test showed good activity of gentamicin-conjugated cements against bacteria and fungi used in this study. Additionally, cytotoxicity results proved that all samples were safe on MG-63 cells up to 50 µg/mL with no more than 7–12% dead cells. From the view of the physico-mechanical properties, bioactivity, biodegradation, and drug release rate, 20BG/β-TCP sample was nominated for practical bone grafting material, where it showed appropriate setting time and a relatively high mechanical strength suitable for cancellous bone.

## Introduction

Bioactive bone cements such as calcium sulfate cements (CSCs)^1^, calcium silicate cements (CSCs)^2^, and calcium phosphate cements (CPCs)^3^ are distinct category of biomaterials that can self-set when mixed with water or an aqueous solution containing phosphate salt. CPCs have attracted increasing attention to be used for bone regeneration when formulated as scaffolds^3,4^. Additionally, the potential to use CPCs as carriers for local and controlled medication delivery is particularly alluring and may be helpful in the treatment of a variety of skeletal disorders, including bone cancers and osteoporosis, which normally need unpleasant and protracted treatments^5,6^.

β-Tricalcium phosphate (β-TCP), a member of the calcium phosphate family, has received a lot of attention as a result of its remarkable bioactivity, appropriate rate of degradation, and similarity to the mineral phase of healthy bone tissue^7,8^. Although β-TCP has the advantages described, its use as a monolithic structure was constrained by its weak mechanical properties. In this regard, a number of researchers attempted to address this shortcoming by mixing β-TCP with an additional polymeric or ceramic ingredient as a novel composite formulation to modify its biological and mechanical features. The therapeutic use of β-TCP is constrained by its poor mechanical performance in bone load-bearing areas^9,10^.

Among bioceramics, bioactive glass (BG) seems like a really promising additive to CPCs because it's biocompatible and capable of releasing important bioactive ions like Si, Ca and P into the microenvironment. Plus, after being inserted into the body, it develops a coating of hydroxycarbonate apatite (HCA), which may connect with the host bone and promote the development of new bone^3,10^. Bioactive glasses can actually promote bond with bone tissue by forming an apatite layer that is similar to the mineral phase found in our bones. This bond can occur when the glasses come into contact with physiological fluids, whether in vivo or in vitro, according to these studies, BG binds to bone more quickly than other biceramics^11^. Some studies showing that incorporating bioglasses into β-TCP can greatly enhance its bioactivity and biocompatibility. The ceramic composite scaffold created by Ma et al., which incorporated β-TCP and magnesium-doped bioactive glass, shown a notable enhancement of the mechanical characteristics, biodegradability, and biocompatibility with respect to the pure β-TCP scaffold^12^. Hesaraki et al. created composites using sol gel technique derived from BG (10, 25, and 40 wt%) and β-TCP. The β-TCP/BG composites showed superior bioactivity, and higher ability to support the growth of human osteoblastic cells^13^. Alumina was added mainly to the glass composition in this study to improve the mechanical strength of the final composite cement^14,15^. Many prior studies on alumina-containing silicate bioactive glass showed that alumina had improved the mechanical characteristics of the glass. In addition, we used alumina in an acceptable percentage according to the glass bioactivity concept. Furthermore, S. Melchers et al.^16^ found that in vitro bioactivity tests exhibit a small decrease in bioactivity upon incorporation of small amounts and a sudden drop beyond 3 mol % of Al_2_O_3_ doping (0.5 to 15 mol%) in ordered mesoporous bioactive glasses (MBGs), based on the composition 80% SiO_2_–15% CaO–5% P_2_O_5_ (in mol%). In another study, β-TCP and β-TCP/Al_2_O_3_ scaffolds were obtained by gel-casting method, and alumina showed great cytocompatibility as there was no cytotoxic and genotoxic effect^17^.

In another study, BG and Sr-dopped—TCP combined to form a composite scaffold was created, and its biological features were evaluated in vitro and in vivo. Results revealed that after being implanted in rabbit models with a critical size defect, the composite scaffold possessed the right mechanical properties for bone tissue engineering as well as bone regeneration^18^. The 45S5 BG has excellent bioactivity in addition to antibacterial activity against some microbes^19,20^. The 45S5 BG was tested by Allan et al. for its capacity to reduce oral bacterial activity that frequently interferes with periodontal defect synthetic repairs^19^. They discovered that 45S5 had a strong antibacterial capacity, which was connected to the bioglass releasing ions into the media, which causes the local physiological pH to change. Six different bioglasses, including 45S5, were tested by Zhang et al. for their ability to inhibit the growth of microbes. For some of the microorganisms they studied that all exhibited antibiotic activity, which they attributed to the medium's increased pH and alkaline ion concentration^20^.

Generally, Ca-phosphate cements (CPCs) have been widely used as drug delivery systems for different active pharmaceutical components. That is because of low exothermic setting reaction of CPCs where most of the drugs are sensitive to the temperature^21,22^. Meanwhile, antibiotics have received the most attention because of their many uses, such as prophylactics to avoid infections caused by surgical treatments and as a general treatment for bone infections^6,23^. The drug can be loaded into the ceramic cements by drug physical adsorption after cement setting or by incorporating it in the cement constituents before preparation by dissolving the drug in the liquid phase for example^24^. And so, drug elution profile depends on the method of drug loading. The drug release can occur by diffusion, desorption, materials erosion mechanism. The drug release kinetic of CPCs is usually diffusion-controlled^25^.

However, fabrication of controlled drug release system based on CPCs is critical. Where either very low or accelerated drug elution is undesirable, so that the drug should be release with a rate that achieve a disease treatment^26^. Accordingly, the drug delivery system can be tailored by adjusting porosity, particle size, incorporation of other materials to obtain composites etc.^27^. The above many prior studies on bioactive glass (BG) seem like a really promising additive to CPCs, in which have focused on the effect of both alkalis and alkaline earth oxides on its structure and properties in the context of improving its bioactivity, biodegradability, and biocompatibility with respect to the pure β-TCP scaffold. In addition to previous literature, reports on the bioactivity and biodegradability properties of β-TCP/BG composites indicate that glass containing Al_2_O_3_ is limited. To the best of our knowledge, borosilicate glass doped with Al_2_O_3_ has not been investigated with TCP to form a composite, and a wide-spectrum gentamicin was also used in this study as a drug model. The drug was loaded into bioglass/β-TCP composite cements.

The main goal of the current study is to tailor the mechanical, biological characteristics, cementation time, and drug elution rate of β-tricalcium phosphate bone cement by adding bioactive glass (BG). BG was varied to obtain cement with optimum properties. This work introduced a potential composite used as an antibacterial bone drug delivery system. Also, the crystalline structure, physico-mechanical, biological, and antimicrobial properties of β-TCP/BG composites were reported.

## Materials and methods

### Preparation of β-tri-calcium phosphate (β-TCP)

The solid state reaction was used to synthesize β-TCP powder. Calcium carbonate (CaCO_3_, 99.00%) and calcium phosphate (CaHPO_4_, 99.00%) were combined for1/2 h in a 2:1 M ratio. All chemicals were provided by BDH Chemicals Ltd, Poole, England. At 1050 °C, this combination was calcined with a heating rate of 5 °C/min and kept for 6 h in an electric oven^17^.

### Preparation of bioactive glass (BG)

The chemical composition of BG that has been melt quenched is given in Table 1. The starting materials of BG were silicon oxide (SiO_2_), calcium carbonate (CaCO_3_), sodium carbonate (Na_2_CO_3_), orthoboric acid (H_3_BO_3_), ammonium dihydrogen phosphate (NH_4_H_2_PO_4_) and aluminum oxide (Al_2_O_3_) powders. From Table 1, the chemical composition of the prepared glass is emphasized. An appropriate amount of the starting materials, which were mechanically homogenized were melted in an electric oven at 1200–1500 °C for 2 h in air using a platinum crucible. To achieve better and continuous homogeneity, every 15 min, the melted substance was turned into the crucible. At room temperature, the melted substance was cooled by putting it into a stainless-steel mold and then moved to another muffle oven and hardened there for 1 h at a temperature of roughly 360 °C. At a rate of 25 °C/h, the muffle was adjusted to cool to room temperature.

**Table 1:** Chemical composition of the prepared BG (wt%).

Glass composition	SiO_2_	CaO	Na_2_O	B_2_O_3_	P_2_O_5_	Al_2_O_3_
BG	50	20	15	7	4	4

### Characterization of the produced samples

The prepared β-TCP and BG samples were ground on an electric milling device made by the German company Retsch GmbH PM100, to get the required particle sizes. After that, X-ray diffraction (XRD) and high-resolution transmission electron microscopy (HRTEM) were used to confirm the configurations of β-TCP and BG. The X-ray diffractometer PW 1730 from Philips was utilized for the XRD investigation. Its Ni-filtered Cu-Kα X-ray radiation (λ = 1.5406 Å) powered was set at 40 kV and 30 mA. HRTEM images were captured using the JEOL JEM-2100 (Japan) microscope, which had a resolution of 1.402 Å and an accelerating voltage of 200 kV.

### Preparation and characterization of the BG/β-TCP composites

The manufactured composite samples contain 100, 95, 90, 85, or 80 weight percent of β-TCP powder and 0, 5, 10, 15, or 20 weight percent of BG powder to achieve a weight percent of 100. Their respective symbols are β-TCP, 5BG/β-TCP, 10BG/β-TCP, 15BG/β-TCP, and 20BG/β-TCP. To create cements with an appropriate consistency for working with, composite samples were mixed with distilled water at a ratio of 0.24 mL/g water to powder. Using a mold with a 10mm diameter and 2 mm height, the produced cements were cast and compressed. After one d of curing in a humidity room with a comparative humidity of around 100% and a temperature of 37 ± 0.5 °C, the samples were demolded.

In order to estimate the setting time, 0.2 g of the freshly manufactured powder were thoroughly blended with one drop of distilled water for 1/2 min before being cast into a mold with dimensions of 10 mm in height and 2 mm in thickness. After leaving the sample for 2 min in the air, the sample was gently lowered to the flat surface of the tested sample using a Gilmre needle (with a 2 mm flat end and a burden of approximately 100 g), it was gently dropped onto the flat surface of the sample being examined. The test was performed for 1/2 min until the sample under test could not be completely circled by the indenter^28^.

Rheology test was carried out on the rotational rheometer MCR 301 (Anton Paar, Graz, Austria) using two parallel plates with a 1 mm gap. The tests were performed at 25 °C ranging from 100 to 0.01 s^−1^^29^.

### In vitro bioactivity and mechanical tests

By soaking the samples in stimulated body fluid (SBF) solution at around 37 ± 0.5 °C (the average body temperature), a biological investigation (In-vitro) was carried out. The SBF solution's chemical makeup and manufacturing process, which is similar to how human blood plasma is generated, elsewhere^30^. To continue the sequence of HA layer production, immersion times in the biological fluid were utilized for 1, 3, 7, 14, and 28 day. Samples were described using the following tools after soaking for a variable amount of time.

A LLOYD device, Model LR 10K, was utilized to study the compressive strength of β-TCP cement and composite sample using the specific method in^31^. At room temperature, the bulk density of composites was calculated using Archimedes' method. Water was employed as the liquid^32^.

In addition to the amounts of released P- and Ca-ions in the biological solution (SBF), changes in pH values were estimated. The pH value had been determined using an electrolyte-type pH meter. ICP-AES, which stands for inductively coupled plasma-atomic emission spectroscopy, was utilized to measure the amounts of Ca- and P-ions.

Scanning electron microscopy (SEM) and (EDS) tests were carried out using a SEM–EDS Inspect S, T810, D8571, FEI Co., Japan) having an acceleration voltage of 30 kV and a magnification of 10 up to 300,000, in order to track the formation of hydroxyapatite layers on the surfaces of the samples prior their immersion in SBF solution.

Additionally, Fourier transform infrared spectra were examined by a Japanese Jasco-300E FTIR spectrophotometer. The synthesize samples were crushed, and the fine powders were combined in a 1:100 ratio with KBr. The mixture was then pressed with a weight of 5 tons per square centimeter to create a clear and homogenous disc. To prevent moisture assault, the FTIR studies were completed right away after the discs were prepared.

### Drug delivery profile and release kinetic

A wide spectrum gentamicin was used in this study as a drug model. The drug was loaded into bioglass/β-TCP composite cements by dissolving gentamicin in the cement liquid phase, and prepared the cements following the previously mentioned method. The drug release profile was studied by soaking 200 mg of composite incorporated drug in 3.5 mL phosphate buffer saline (PBS) at pH 7.4^33^ up to 7 day. 1 mL of incubation solution was collected at each predetermined time (3, 6, 12 h, 1, 2, 3, 5, 7 day) and kept at – 20 °C up till the measurement. The same volume of fresh PBS was added instead of the taken solution. Standard curve of the drug was made by preparation of known concentrations of the drug and measured their absorbance by UV/VIS spectroscopy at wavelength 232 nm. This standard curve was used to determine unknown drug concentration in the collected release solutions.

The elution mechanism of gentamycin loaded into the ceramic cements was studied by using various kinetic models, such as first order, Higuchi, Hixson–Crowell, and Baker–Lonsdale models, to determine, approximately, the rate and way of drug released from such cements. Various kinetic models are represented by the equations below:

The relationship between the cumulative proportion of drug released and the square root of time is known as the Higuchi model^34^. The release of water-soluble and weakly soluble from a range of matrices, including solids and semi-solids, can be studied using this model in Eq. (1).

1 $${\text{C}}_{{\text{t}}} = {\text{ K}}_{{\text{H}}} {\text{t}}^{{{1}/{2} }}$$

The Baker–Lonsdale model^35^ was developed from the Higuchi model by Baker and Lonsdale (1974) and it characterized the drug release from spherical matrices using Eq. (2):

2 $${3}/{2}\left[ {{1} - \left( {{1} - {\text{C}}_{{\text{t}}} } \right)^{{{2}/{3}}} } \right] - {\text{C}}_{{\text{t}}} = {\text{ K}}_{{{\text{BL}}}} {\text{t}}$$

Korsmeyer–Peppas model is represented by Eq. (3):

3 $${\text{C}}_{{\text{t}}} /{\text{C}}_{0} = {\text{ K}}_{{\text{K}}} {\text{t}}^{{\text{n}}}$$

where, the model is fitted on the first 60% of drug release data. Where C_t_ is the amount of drug released in time t, C_0_ is the initial amount of drug in the sample, K_h_, K_b,_ and K_k_ are rate constants dervied from Higuchi, Baker-Lonsdale, and Korsmeyer–Peppas models, respectively, and n is the kinetic exponent. Korsmeyer–Peppas model depends on determination of “n” value to find out the mechanism of drug release^36^. Where, the release is considered a Fickian diffusion mechanism when n = 0.45, and it is non-Fickian transport in cases of 0.45 < n < 0.89, and when n = 0.89 the release is Case II (relaxational) transport, and in case of n > 0.89 it is super case II transport. The kinetic exponent, n, can be determined by plotting of log cumulative percentage of drug release versus log time. The regression coefficients, R^2^, were calculated to judge how well the data match the kinetic model.

### Antimicrobial investigation

Antimicrobial ability of the designed materials combined with gentamicin and alone was evaluated using microbial pathogens those kindly obtained from Microbiology and Immunology Dep., Faculty of medicine (Boys), Al-Azhar University. One Gram-positive bacterial strain (*Staphylococcus aureus*), two Gram-negative bacterial strains (*Pseudomonas aeruginosa*, and* Klebsiella pneumonia*), one unicellular fungal strain (*Candida albicans*), and one multicellular fungal strain (*Mucor racemosus*) were pre-activated using Nutrient broth (for bacterial pathogens at 37 °C for 24 h) and Potato Dextrose broth (for fungal pathogens at 28 °C for 48 h). Inoculum size of each pathogen was justified to be constant at 10^6^/mL approximately, spore suspension was then dispensed over the agar plate medium and circle pore was marked and the tested material was poured with different samples^37^. Moreover, screening of each of ceramic-loaded gentamicin and unloaded ceramic was performed at different concentration of ceramic and fixed gentamicin concentration. Incubation of each tested pathogen was carried out and the observed results were recorded in three replicates and compared to the standard antibacterial and antifungal agents. The observed differences between the Ceramic-loaded Gentamicin and unloaded Ceramic were evaluated based on the inhibition zone diameter (mm) around each pathogen^38^. Antibacterial and antifungal agents were compared to the tested materials efficiency.

### Cytotoxicity test

#### Cell culture

The human bone osteosarcoma cell line (MG-63) was provided from ATCC, USA. The culture was maintained in Dulbecco's Modified Eagle's Medium (DMEM), which was enhanced with 10% fetal bovine serum (FBS), 2 mM l-glutamine, and 100 units/mL of penicillin G sodium, 100 units/mL of streptomycin sulphate, and 250 ng/mL of amphotericin B. The cells were kept in humidified air with 5% CO_2_ at sub-confluency temperature of 37 °C. When confluence reached 75%, cells were used. The test samples were suspended in 10% sterile dimethyl sulfoxide (DMSO) and diluted in the medium before being added to the cells at the assay-specified concentration (100–3.125 µg/mL). All of the materials used in cell culture were purchased from Cambrex BioScience, located in Copenhagen, Denmark. All chemicals were obtained from Sigma/Aldrich, USA, except those mentioned. All experiments were repeated four times, unless mentioned.

Cytotoxicity of the tested samples was assessed against the MG-63 cell line using the MTT Cell Viability Assay. MTT (3-[4,5-dimethylthiazole-2-yl]-2,5-diphenyltetrazolium bromide) assay is the ability of live cells' active mitochondrial dehydrogenase enzyme to cleave the yellow MTT's tetrazolium rings and form dark blue, insoluble formazan crystals that accumulate within healthy cells because they are mostly impermeable to cell membranes. Crystals are liberated by solubilization of the cells, which leads to their further solubilization. The amount of soluble formazan dark blue color is strongly correlated with the quantity of live cells. By measuring the absorbance at 570 nm, the amount of MTT reduction was determined^39^.

#### Reagents preparation

MTT solution: 5 mg/mL of MTT in 0.9% NaCl.

Acidified isopropanol: 0.04 N HCl in absolute isopropanol.

#### Procedures

Cells (0.5 × 10^5^ cells/well), in serum-free media were plated in a flat bottom 96-well microplate and treated with 20 µL of various doses of the tested samples for 48 h at 37 °C, in a humidified 5% CO_2_ atmosphere. The range of concentrations utilized was 100 µg/mL to 3.125 µg/mL. Following incubation, the media were taken out and 40 µL of MTT solution per well were added. Incubation was then continued for four hours. Following the solubilization of MTT crystals with 180 µL of acidified isopropanol per well and plate shacking at room temperature, the absorbance at 570 nm was photometrically determined using a microplate ELISA reader (FLUOstar OPTIMA, BMG LABTECH, Ortenberg, Germany). Each concentration was repeated three times, and the average was determined. The data were presented as a percentage of relative vitality between the vehicle control and untreated cells, where cytotoxicity was denoted by a relative viability of less than 100%.

#### Calculation

Equation (4) was used to calculate the relative viability percentage

4 $$\frac{\mathrm{Absorbance \, of \, treated \, cells}}{\mathrm{Absorbance \, of \, control \, cells}}\times 100$$

Then the half-maximal inhibitory concentration (IC_50_) was calculated from the equation of the dose response curve. The antibacterial and cytotoxicity suggestions are EXEMPT from the ETHICAL REVIEW with the number No.: EX0050072023.

### Statistical analysis

The P values < 0.05 were established as statistically significant, and all data were presented as mean standard deviation (SD) for n = 3.

## Results and discussion

### Materials characterization

**Figure 1:**
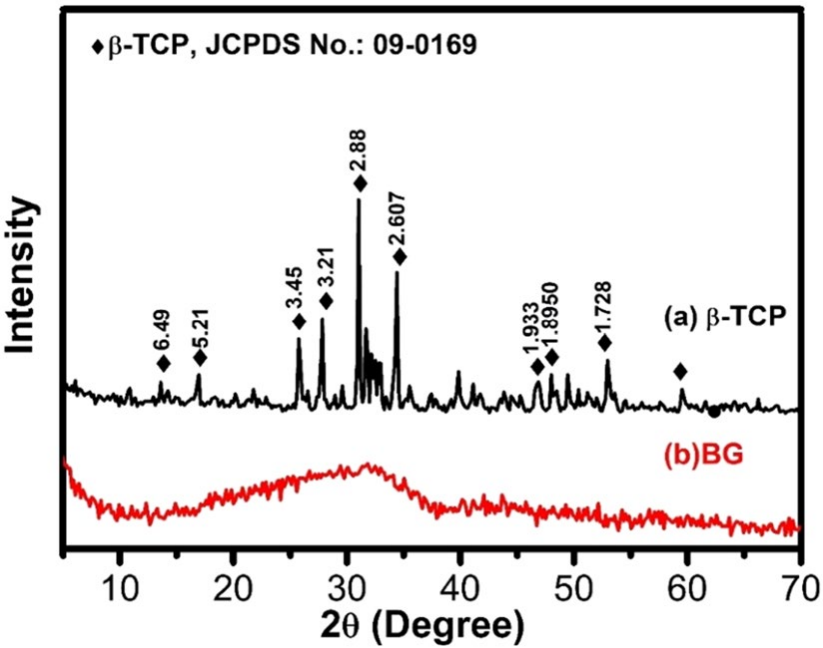
XRD patterns of the prepared (**a**) β-TCP and (**b**) BG.

**Figure 2:**
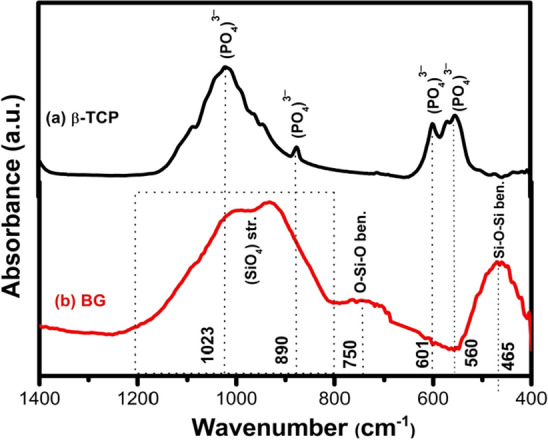
FTIR spectra of the prepared (**a**) β-TCP (**b**) BG.

Figure 1a shows XRD pattern of β-TCP. As shown from the figure, all characteristic diffraction peaks of crystalline β-TCP (JCPDS No. 09-0169) were observed which confirmed that the prepared ceramic was entirely pure β-TCP without additional formed phases. Meanwhile, there were no diffraction peaks detected for BG (Fig. 1b) which proved the amorphous structure of bioactive glass. The FTIR spectra of β-TCP and BG are shown in Fig. 2a and b, respectively. The spectrum of BG showed main bands at 465 cm^−1^ and 750 cm^−1^ attributed to Si–O–Si bending vibration for O–Si–O and bending of orthosilicate SiO_4_^4−^, respectively. Strong and wide band in the range 800–1200 cm^−1^ was assigned to stretching vibration of silicate units in glass structure^40^. Sharp bands in β-TCP spectrum was related to asymmetric elongation connection *v*_4_^−^ (*PO*_4_^3−^) at 602, 560 cm^−1^ and asymmetric stretching band *v*_3_^−^ (*PO*_4_^3−^) at 1023 cm^−1^. The band near 900 cm^−1^ displayed by the spectrum was related to the asymmetric vibration modes of the P–O–P^41^.

**Figure 3:**
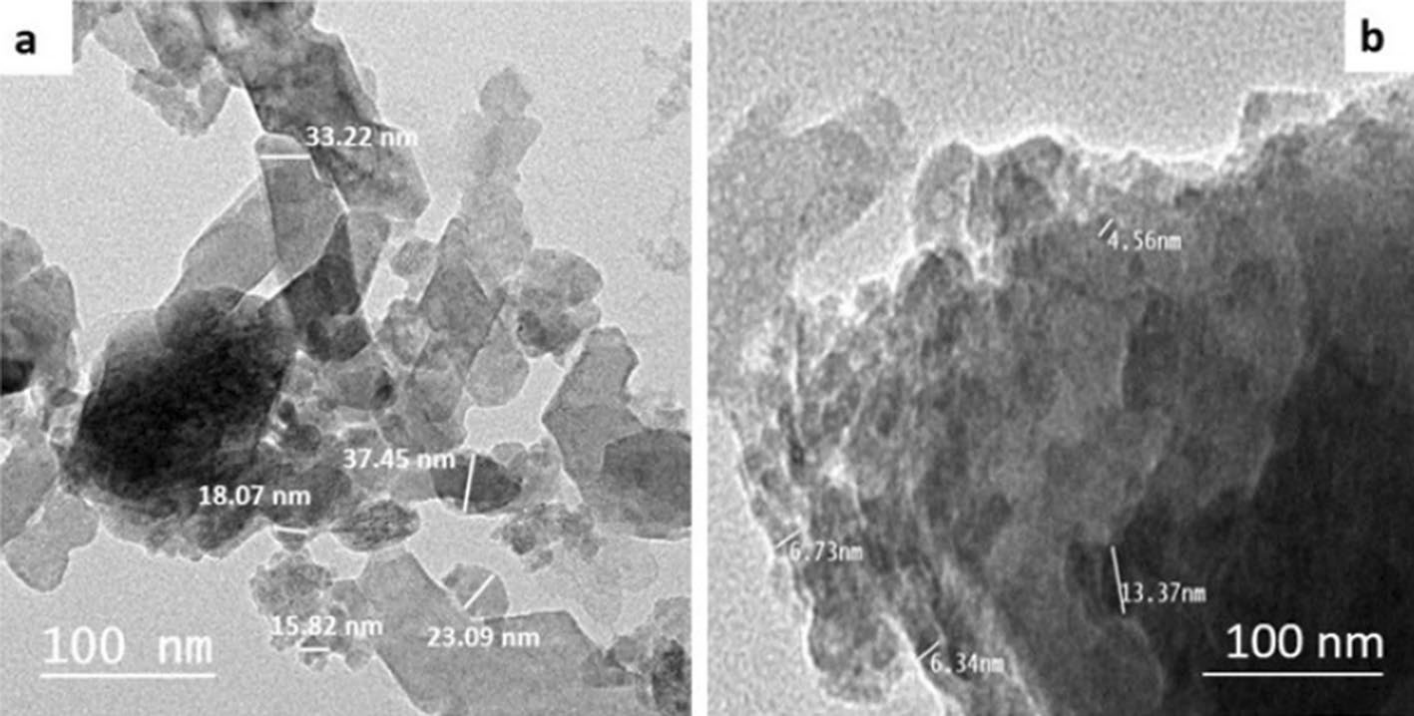
Transmission electron photomicrograph of (**a**) β-TCP and (**b**) BG.

Moreover, the particle size of the prepared materials was investigated by TEM. Figure 3 presents TEM images of β-TCP and BG, where the particle size range of β-TCP was 16–38 nm, while the range of particle size of BG was 5–13 nm. The glass and β-TCP particles possessed subhedral and anhedral morphology.

### Setting time

**Figure 4:**
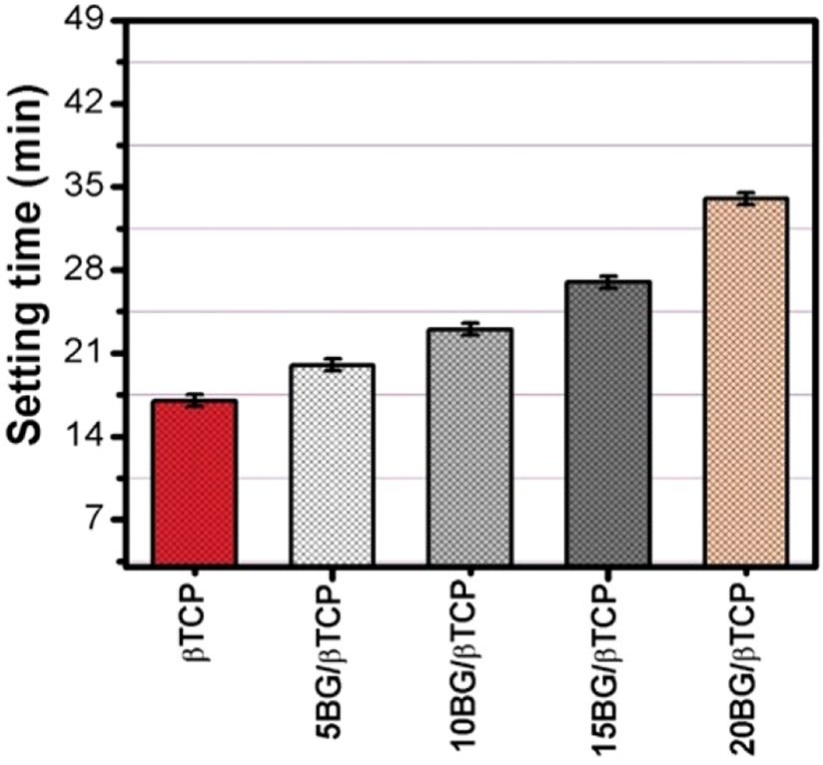
Setting time values of BG/β-TCP composites.

Determination of final setting time of biocements is very important in clinical applications^42^. The final setting time is known as the time needed for cement hardening to a defined consistency^43,44^. The appropriate working time for injectable bone cement in clinical applications should be 20 min or less. Figure 4 shows the setting times of BG/β-TCP composites. The results presented that the setting time of BG/β-TCP composits increased as the BG content increase. It increased from 17 for β-TCP sample to 34 min. for 20BG/βTCP sample. However, the obtained setting time of the composite cements exceeded the clinical requirements, but scientists stated that the prolonged setting time is helpful in some cases to provide extra time in complicated surgeons require more time^3,45,46^.

### Rheological property

**Figure 5:**
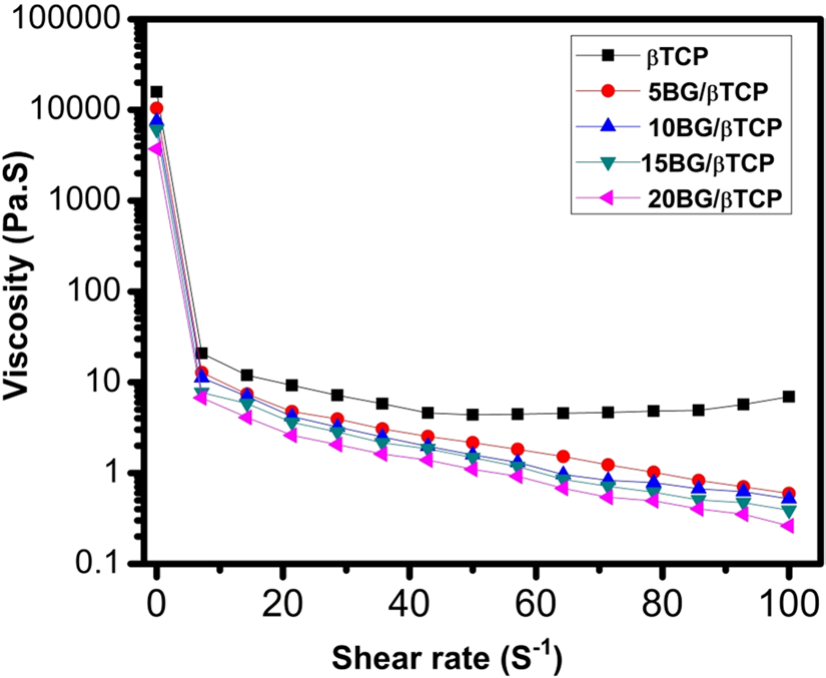
Viscosity of BG/β-TCP composite samples versus shear rate.

The variation of viscosity (Pa.S) with shear rate (S^−1^) is shown in Fig. 5. From the figure it can be noticed that all cement pastes followed shear-thinning behavior (in which the viscosity decreases as shear rate increases). This indicated a probable good injectability of these cement pastes. Moreover, it was clear that adding BG to the cement pastes reduced their viscosities, especially when BG content was high. A surgeon's ability to extrude cement through a nozzle or insert prosthesis into the cement mass will be more challenging the higher the viscosity of the bone cement. Yet, when bone cement is used to secure prosthesis, its low viscosity state allows it to penetrate through the trabeculae of the bones. The penetration facilitates a stronger interlock between the cement and the bone, hence reducing the likelihood of a failure at the interface between the two materials^29^. Setting time results support these findings.

### Compressive strength

**Figure 6:**
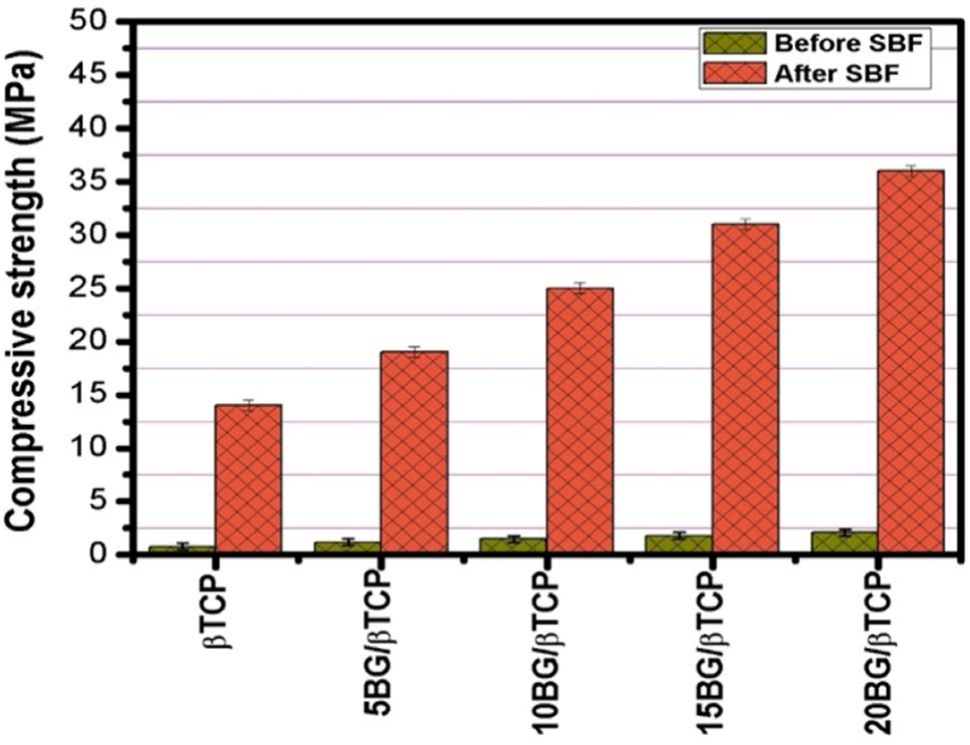
Compressive strength values of BG/β-TCP composite samples prior and after immersion in SBF for 28 d.

Compressive strength of BG/β-TCP composites was tested before and after SBF treatment as can be illustrated in Fig. 6. Before SBF-soaking, the samples showed very low compressive strength with a small increase in BG additions from 0.8 MPa (0% BG) to 2.1 MPa (20% BG). The increase in strength can be due to the nanocomposite materials which dispersed in the cement matrix could exert size-related reinforcement effects and consequently improves the mechanical properties of composites^3,46,47^.

However, immersion in SBF for 28 day at 37 °C significantly improved the strength of all the composites, and the increase in the BG addition gradually increased the strength value to 14 MPa for pure β-TCP to 36 MPa for 20% BG/β-TCP. This can be assigned to enhancement of apatite layer formation by BG. When BG immersed in an aqueous solution the Si–O–Si bridging oxygen breaks and form SiO_2_^−^ groups, and hence the cement surfaces become rich in this layer. This layer attracts calcium and phosphate ions from the solution providing nucleation sites for apatite crystal formation. In the case of pure β-TCP, The Ca^2+^ and PO_4_^3−^ ions need to be supersaturated in surrounding solution in order for apatite to form, which needs more time to establish an apatite layer on the cement surface^48,49^. Moreover, BG incorporation into β-TCP forms a dense and homogeneous cement microstructure due to the mesoporous BG nanoparticles surrounding the TCP particles in a way that fills in cement matrix, which improves the composite's mechanical properties^3^. These mechanical strength values were relatively high compared to the cements prepared in previous studies and related to the cement obtained in this study. In earlier research, Zhang et al.^50^ demonstrated the maximum compressive strength of BG/β-TCP scaffold, reaching a value of 22.12 MPa. Pu et al.^51^ found that after 5 wt% 75S25C BG addition, the compressive strength of CPC increased from 21.52 MPa to 30.17 MPa. Bose et al.^52^ indicated that a 5 wt% BG in TCP composite shows a compressive strength of 26.7 MPa for random porosity structures having a total porosity of ~ 47.9%. Mesoporous BG particles were added to the CPC by El-Fiqi et al.^49^, the compressive strength increased from 12 to 26 MPa of the final CPC composite.

As we mentioned before, further increase of BG amount inside the CaP phase led to the reduced strength of the composite^51,53^. Later on, authors examined bioactivity by the morphological observation of BG incorporated CPC composites with the aim to identify the compressive strength fall. Their results indicated that increased BG content could be destructive for the bonds among the CPC crystals and polypeptide poly (γ-glutamic acid) which was presented in their liquid phase^51,53^.

### Bulk density and apparent porosity

**Figure 7:**
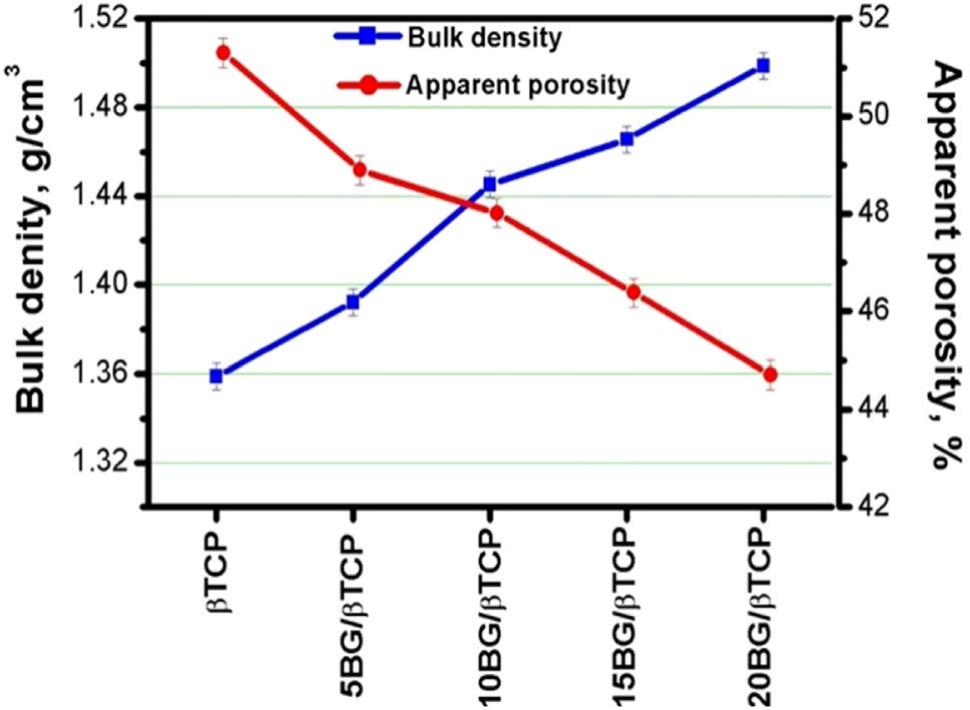
Bulk density and apparent porosity values of BG/ β-TCP composite samples after immersion in SBF for 28 d.

The bulk density and apparent porosity of the composite cements were evidently affected by addition of BG to β-TCP after immersing in SBF up to 28 day (Fig. 7). Bulk density of the composite cements (BG/β-TCP samples) was higher than that of β-TCP cement sample. However, the apparent porosity was inversely proportional to bulk density, and so it decreased for BG/β-TCP composites. This can be explained by the BG role to slow down of setting reaction of the composite cements. Where, slow cement setting leading to form condensed structure and dependly low apparent porosity^54^.

### Alteration of pH and Ca, P ionic concentrations

**Figure 8:**
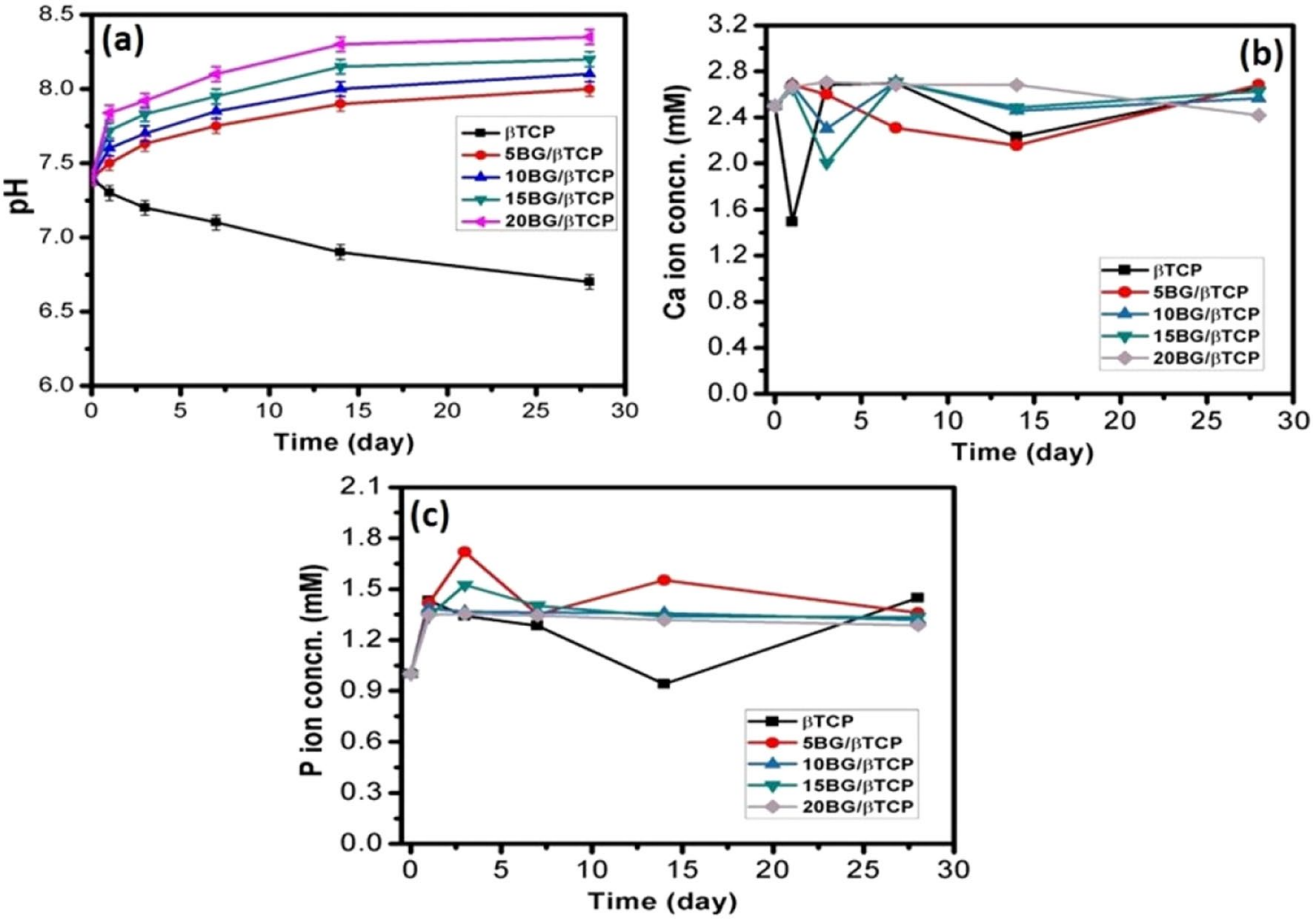
Variation of (**a**) pH, (**b**) Calcium ion conc. and (**c**) Phosphate ion conc. against soaking time in SBF solution for BG/β-TCP composite samples.

Figure 8a explains the pH alteration during soaking of β-TCP and BG/β-TCP composits in SBF solution. The pure β-TCP caused a decrease in pH from 7.4 to 6.7 from 1 to 28 day of soaking time. Whereas, the entire prepared BG/β-TCP composites induced an increase in pH during the same time period. The drop and continual decrease in pH in case of the pure β-TCP was likely ascribed to formation of phosphoric acid (H_3_PO_4_) during hydration of β-TCP to hydroxyapatite according to the following equation^55,56^. Meanwhile, presence of BG particles in the cements neutralized this acidic reaction due to release of alkaline ions from the glass. This neutral pH is advantageous to diminish or prevent inflammatory response of the body to acidic environments^57^.

$${1}0{\text{ Ca}}_{{3}} \left( {{\text{PO}}_{{4}} } \right)_{{2}} + {\text{ H}}_{{2}} {\text{O}} \to {\text{Ca}}_{{{1}0}} \left( {{\text{PO}}_{{4}} } \right)_{{6}} \left( {{\text{OH}}} \right)_{{2}} + {\text{ 2 H}}_{{3}} {\text{PO}}_{{4}}$$

Figure 8b and c show ion release behavior of calcium and phosphate ions of pure β-TCP and BG/β-TCP composite specimens at different soaking time. It can be observed from the figure that Ca^2+^ ion concentration was abruptly decreased in the initial time in the solutions incubated β-TCP, 10BG/β-TCP, and 15BG/β-TCP samples followed by gradual decrease stage. While, it initially increased in case of 5BG/β-TCP and 20BG/β-TCP samples. However, the concentration decreased gradually for 5BG/β-TCP sample; meanwhile it became nearly constant for 20BG/β-TCP sample after this initial increase. On the other hand, phosphate ions concentration was increased after 1 day of soaking time for all cements that was because of formation of phosphoric acid (H_3_PO_4_) during hydration of β-TCP to hydroxyapatite, as mentioned above. The concentration became approximately constant in solutions incubated 10BG/β-TCP, 15BG/β-TCP, and 20BG/β-TCP cements, while the concentration was fluctuated for β-TCP and 5BG/β-TCP samples. This change of ion concentrations throughout the soaking time was attributed to formation of hydroxyapatite layer on the cement surfaces. Meanwhile, the released ions have an important role in accelerating cement hardening and apatite formation. Addition of BG particles to β-TCP cement could permit forming of silanol layer, which imparts favorable sites for nucleation of apatite bone-like layer which mainly depends on the supersaturating of body fluid with calcium and phosphate ions. Addition of network modifiers such as Ca^2+^ and Na^+^ to bioactive glass assists dissolution of the glass and formation of non-bridging oxygens (NBOs). Where, release of Na^+^ ions into the SBF via an exchange with the H^+^ (or H_3_O^+^) ions from the fluid to form Si–OH (silanol) groups on the glass surface. These groups combine with calcium and phosphate ions from the SBF to form an amorphous calcium phosphate. Once the apatite nuclei are formed, they could spontaneously grow by consuming the calcium, phosphate and OH^−^ ions from the SBF recrystallize to bone-like apatite crystals thereafter^58^. As mentioned before, alkali ions released from the BG reverse the effect of phosphoric acid produced during hydration of β-TCP as illustrated in Fig. 8a ^51,59^.

### FTIR spectroscopy

**Figure 9:**
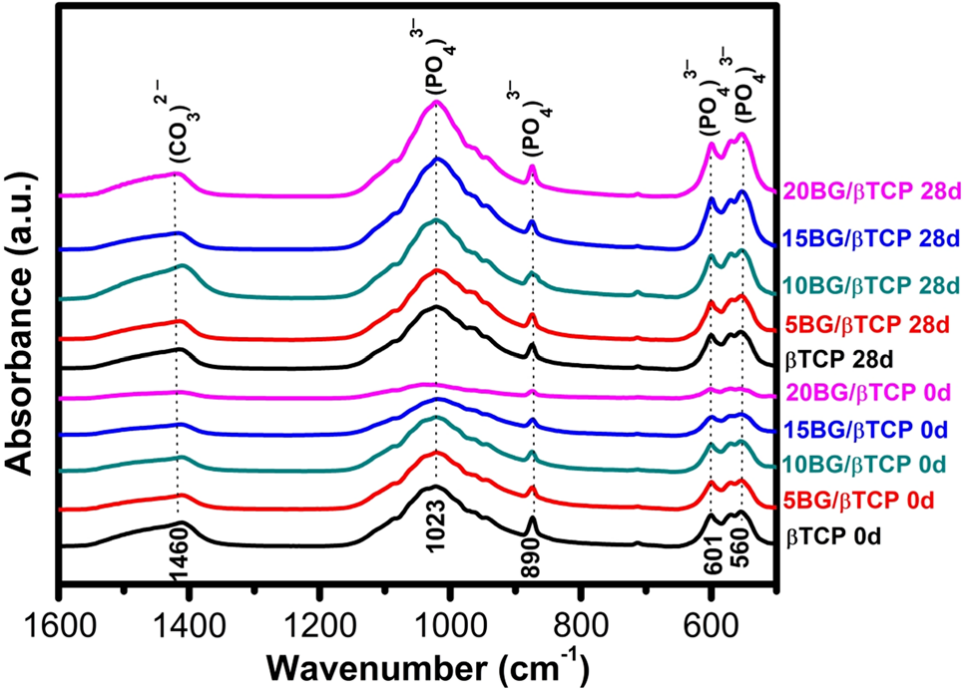
FTIR spectra of studied BG/β-TCP samples before (0 day) and after (28 day) immersion in SBF solution.

Figure 9 displays the Fourier transform IR spectra patterns of prepared samples before and after soaking in SBF solution. FT-IR spectra of pure β-TCP cement before soaking in SBF solution showed sharp bands related to asymmetric elongation connection *v*_4_^−^ (*PO*_4_^3−^) at 602, 560 cm^−1^, the band near 900 cm^−1^ was related to the asymmetric vibration modes of the P–O–P, and asymmetric stretching band *v*_3_^−^ (*PO*_4_^3−^) at 1023 cm^−1^^41,60^ in addition to carbonate groups (CO_3_^2−^) band that are observed at 1460 cm^−1^ due to a very rapid carbonation occurs on the cement surface when it in contact with air^56,61^. It was observed that these bands decreased with an increase in BG addition. The FTIR spectra pattern of BG/β-TCP composite samples after soaking in SBF for 28 day. As it can be observed in this spectra, both bands of P–O bend and P–O stretch, which all belong to phosphate groups of hydroxyapatite^56,60^ become sharper with increasing introduction of BG in TCP cement, as they were elucidated from the calculation of area under peak, which were 3.17, 3.17, 3.70, 4.90, and 5.56 for β-TCP, 5BG/β-TCP, 10BG/β-TCP, 15BG/β-TCP, and 20BG/β-TCP, respectively. Thus, FT-IR is a leading evidence to suggest that BG addition significantly enhances TCP’s dissolution/precipitation rate^49,62^.

### X-ray diffraction

**Figure 10:**
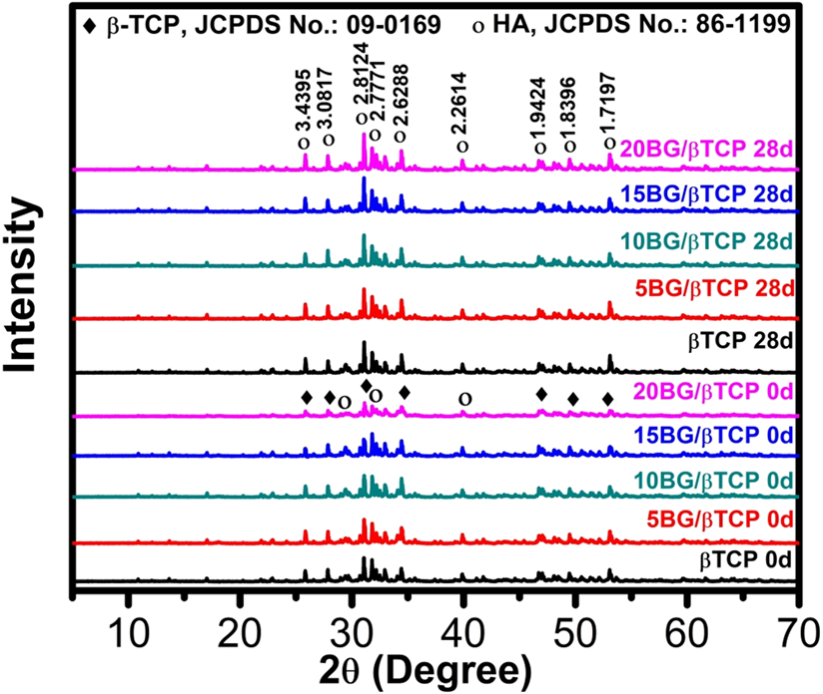
XRD patterns of prepared samples before (0 day) and after (28 day) immersion in SBF.

The structural crystalline phase of as-prepared β-TCP and BG/β-TCP composite cements before and after soaking in SBF solution was determined by XRD analysis (Fig. 10). The XRD patterns identified β-TCP before immersion in SBF solution as a main crystalline phase as shown from the characteristic diffraction peaks at 2θ of 31.02°, 34.37°, 27.76°, 52.94° (JCPDS card no. 09–0169) with minor amount of hydroxyapatite phase observed at 2θ of 31.79°, 32.92°, 39.83° (JCPDS card no. 86–1199). Hydroxyapatite phase existence can be attributed to the initial setting of β-TCP in which β-TCP was partially hydrated to hydroxyapatite. By increase of bioactive glass content in the composite samples, the diffraction peak intensities decreased^49,55^.

The soaked samples in SBF for 28 day showed remarkable crystal changes compared to those before soaking. The XRD spectra illustrated the development of the characteristic hydroxyapatite diffraction patterns which are at 2θ of 31.79°, 32.92°, 39.83° and following the JCPDS Card No. 86–1199. It can be observed that apatite peak intensities of the 20% BG cement appeared to be stronger than those of pure β-TCP due to the formation of more precipitated apatite^47,60^. XRD pattern of composites cements (BG / β-TCP) revealed greater peaks of hydroxyapatite phase intensity compared to β-TCP cement, showing higher production of hydroxyapatite (HA), and this supported their better bioactivity^49,56,63^.

### SEM micrographs photos

**Figure 11:**
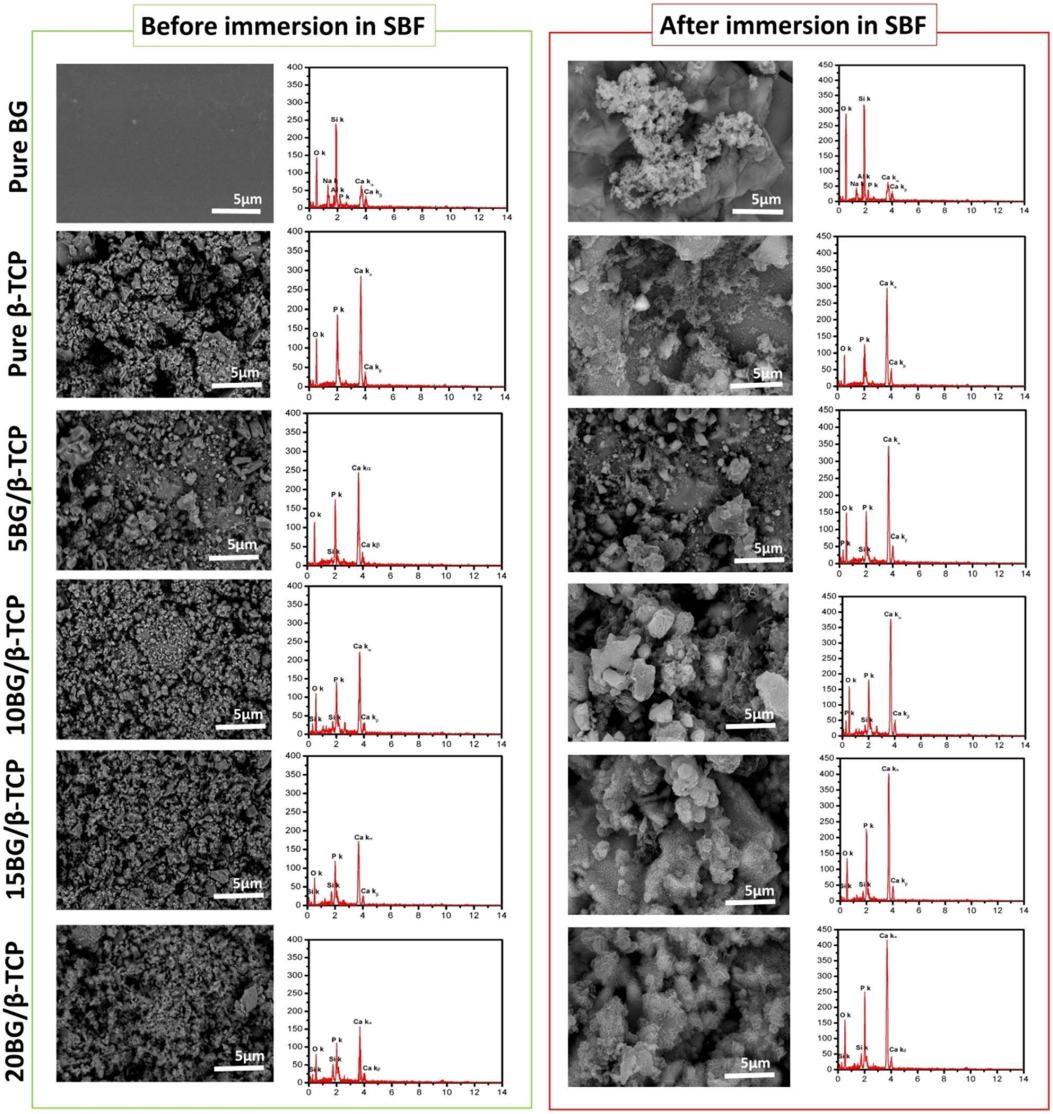
SEM images coupled with EDS spectra of BG, β-TCP, 5BG/β-TCP, 10BG/β-TCP, 15BG/β-TCP, and BG/β-TCP before and after immersion in SBF solution for 28 day.

Figure 11 shows SEM coupled with EDS spectra of different samples before and after immersion in SBF for 28 day. The SEM/EDS of the pure BG before and after soaking in SBF presented that the glass-surface soaked in SBF possessed noticeable changes in the surface microstructure compared to unsoaked glass. A porous particulate apatite layer can be noticed on the glass immersed in SBF. This layer formed, as mentioned before, by the chemical reaction between BG surface and the physiological solution^64^. Some cracks were observed throughout newly formed apatite layer which probably formed by means of contractions in the porous hydrated layers when the specimens were dried^64^. Formation of apatite layer can be confirmed by EDS spectra, where Ca/P increased considerably after BG immersed in SBF (1.5) compared to that did not immersed in SBF (3) referring to formation of hydroxyapatite (HA) layer. The SEM/EDX of β-TCP sample previous soaking in SBF showed only Ca and P atoms, whereas SEM/EDS of composites cements presented Si beside Ca and P atoms which is ascribed to BG. EDS observations support SEM images and consistent with above XRD analysis^55^. After immersion in SBF, the SEM photos of β-TCP and BG/β-TCP composites demonstrated crystals with plate-like morphology which is characteristic to HA crystals. As referred before, HA was formed due to hydration of β-TCP to HA (as confirmed by XRD). Comparatively, massive amounts of HA spherule aggregates covering their plate-like crystalline surface were observed on the surface of BG/β-TCP composites. The surface of 20BG/β-TCP composite (contained the highest percentage of BG) was covered with more abundant spherical apatite crystals than the other composite samples, and EDS spectra showed extremely raise in the ratio of Ca/P peaks (1.64). Therefore, high content of BG significantly improved the bioactivity and mechanical properties of the final cements, and hence, 20BG/β-TCP composite exhibited more promising material for interaction with bone cells as compared to β-TCP used alone.

### Drug delivery

**Figure 12:**
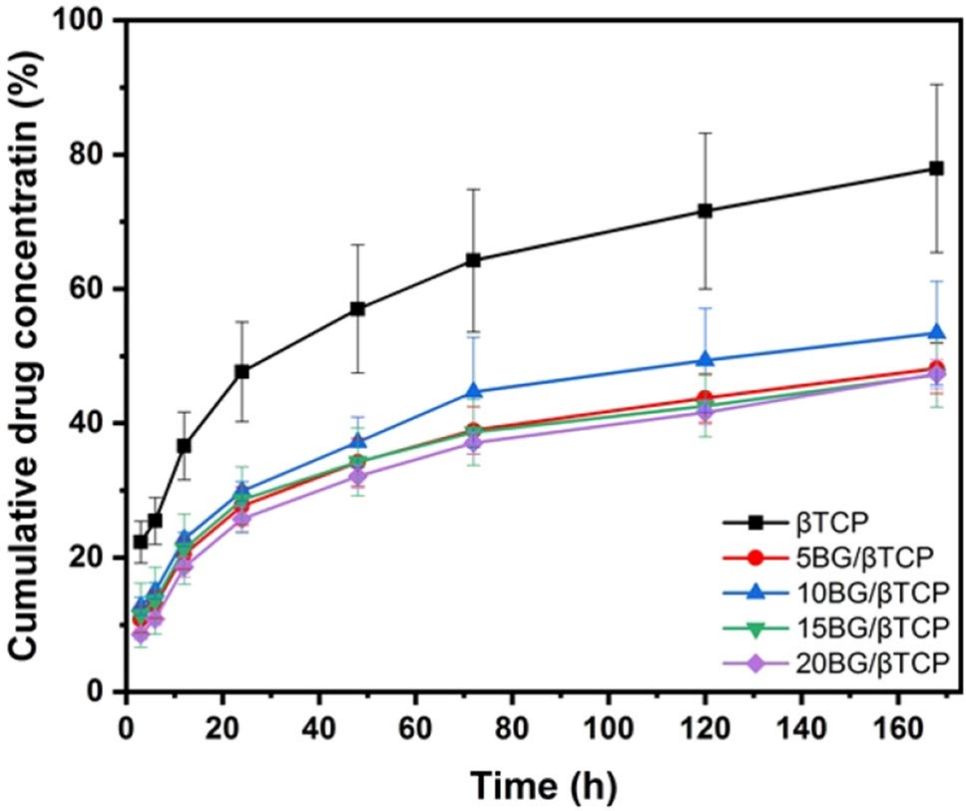
Cumulative drug concentration (%) released from β-TCP, 5BG/β-TCP, 10BG/β-TCP, 15BG/β-TCP, and 20BG/β-TCP samples.

Figure 12 represents the in vitro gentamicin release of β-TCP, 5BG/β-TCP, 10BG/β-TCP, 15BG/β-TCP, and 20BG/β-TCP samples during 7.4 day of immersion in PBS at pH 7. It could be observed from the figure that all samples demonstrated the same sustained drug release behavior. Where there were two stages of release; initial fast release followed by slow release. Fast release stage occurred from instant soaking to 24 h. The percentage of drug released from β-TCP was larger than that released from other composite cements. After first day of immersion 47.7%, 27.7%, 29.9%, 28.7%, and 25.7% was released from β-TCP, 5BG/β-TCP, 10BG/β-TCP, 15BG/β-TCP, and 20BG/β-TCP samples, respectively. Slow release stage followed the fast release stage up to the end of immersion period, and the percentage of drug released at the end of soaking time was 78%, 48%, 53%, 47%, and 47%, respectively. From these results it can be noticed that the composite cements retained nearly 50% of the drug, and so, addition of bioactive glass to β-TCP decreased drug release rate. This can be explained from two aspects. The first one is cement physical properties. As mentioned before, an increase of glass percentage decreased the cement porosity which decreased a chance of drug to release freely from the composites cements. The second reason was likely formation of hydrogen bonding between the drug and glass particles. Where, gentamicin molecule possesses OH groups, and silicate glass when immersed in an aqueous solution can form Si–OH and P–OH groups on its surface, therefore, hydrogen bonding can be generated between glass surface and drugs^65,66^. It was noted that drug release did not decrease linearly with an increase in glass content, which was most likely due to the interference of these two factors. Thus, gentamicin release from β-TCP can be tailored by addition of bioactive glass and achieved slower drug release for an elongated period to prevent bacterial re-infection^67^.

Determination of drug release kinetics is important to know the mechanism and rate of drug release. Different models applied to the obtained drug release data, but the most fitted models (indicated by the correlation coefficient, R^2^) with those data were Higuchi, Baker–Lonsdale, and Korsmeyer–Peppas models. Table 2 represents the release constants and correlation coefficients of such models. From the table it can be noticed that the in vitro drug release rate was drastically decreased by addition of BG. Moreover, all studied cement samples showed a relatively high linearity with Baker-Lonsdale model, where, R^2^ ranged from 0.995 to 0.999. This indicated that the mechanism of drug release from the cements was a non-Fickian diffusion release from spherical particles. This non-Fickian release was likely arised from formation of hydroxyapatite crystals on the cement surface.

**Table 2:** The release constants and correlation coefficients of the Higuchi, Baker–Lonsdale, and Korsmeyer–Peppas models.

	Higuchi		Baker–Lonsdale		Korsmeyer–Peppas	
	K_h_	R^2^	K_b_	R^2^	n	R^2^
βTCP	5.005	0.958	0.298	0.999	0.648	0.985
5BG/βTCP	3.354	0.958	0.389	0.995	0.769	0.979
10BG/βTCP	3.731	0.964	0.279	0.996	0.747	0.986
15BG/βTCP	3.173	0.947	0.284	0.999	0.726	0.974
20BG/βTCP	3.425	0.962	0.284	0.999	0.863	0.975

### Antimicrobial results

**Figure 13:**
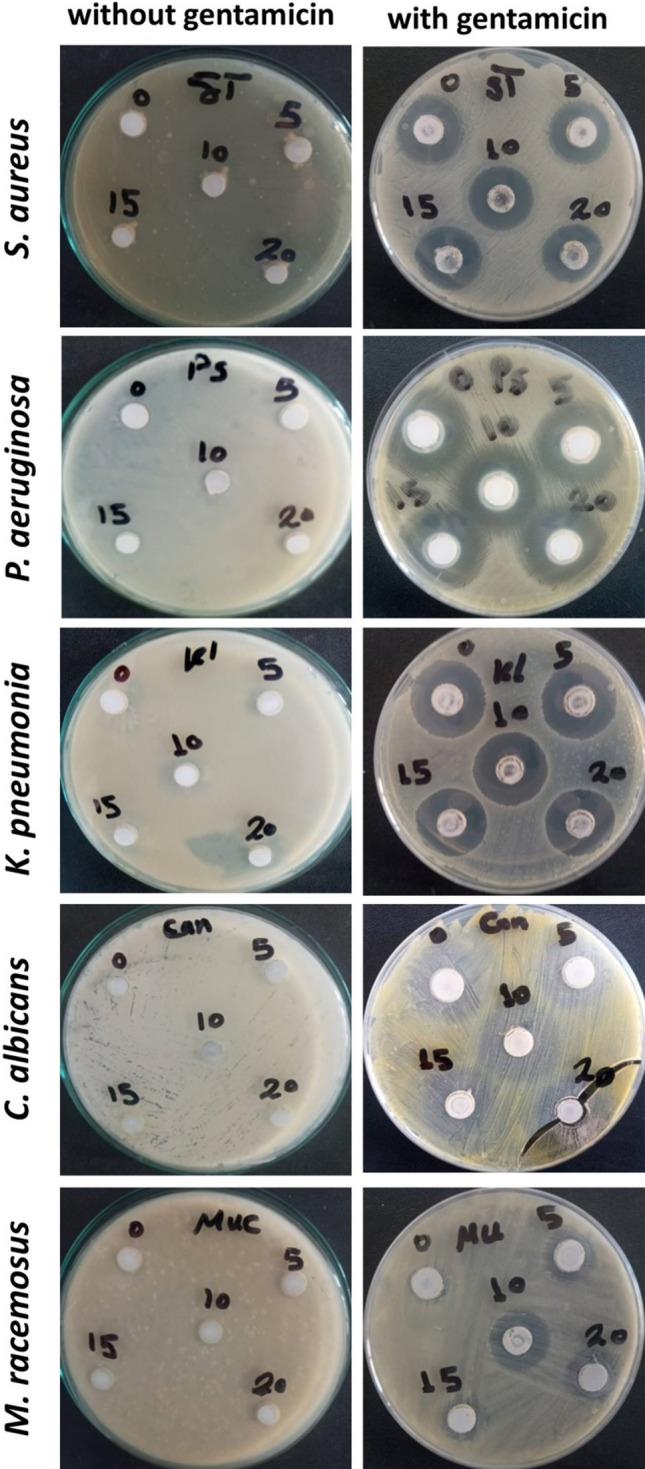
Antimicrobial activity of different cements mixed with gentamicin compared to unmixed ones using agar-well diffusion.

Inefficient of bioceramic materials to prevent the microbial infection still displayed a big barrier toward various bone and wound surgeries. Usually, the bacterial infection to be more active and transferred throughout these materials reflected there have not any antimicrobial activity. Therefore, it’s more important to adding antimicrobial agent to these materials in order to avoid their role as spore-carrier and caused harmful diseases. Accordingly, selection of potent antimicrobial agent to combine with these materials could be to provide a promising protection from microbial attacks is more desirable. For this purpose, preparation of different cements was achieved in the presence of gentamicin and evaluation of the antimicrobial susceptibility was carried out against different standard microbial pathogens. As shown in Fig. 13 and Table 3, the weakness of ceramic cements to prevent the microbial infection was certainly observed for each tested strains reflected their suitability for microbial growth. Therefore, owing to its defect, antibiotics was consider as a significant factor to gained these materials antimicrobial activity in order to be useful for the medical application. The efficiency of the cement-loaded gentamicin was clearly indicated particularly against bacterialpathogens. Otherwise, the ability of cement-gentamicin to prevent the fungal proliferation was found also to be active at a high concentration of cement material. On the other hand, homogenous combination between the composite cements and gentamicin was proved via the greatest inhibition zone around each concentration of ceramic, since each concentration of cement become valuable toward *Pseudomonas aeruginosa*, *Klebsiella pneumonia*, and *Staphylococcus aureus.* In addition, fungal strains was found to be more affected 10BG/βTCP and 20BG/βTCP samples (i.e. at high percentage of BG) emphasized an excellent homogeneity between them in these concentration against multicellular fungi like *Mucor racemosus.* Furthermore, the tested bacterial pathogens were characterized as B-lactamase producers, which was resistance to the first generation cephalosporin such as Cephradine and Cephalexin. Meanwhile, *Mucor racemosus* was able to resistance antifungal group, Azole such as Fluconazole. Thus, the promising results against both bacterial and fungal pathogens open a new era to protect the ceramic materials from microbial infections, particularly those having a capability to resistant different classes of antibiotics. Meanwhile, the reaction of cement process did not affect the bioviability of gentamicin in all samples, and drug release rate can be tailored by addition of BG to β-TCP (as shown from drug delivery results), as well as, the samples showed good in vitro bioactivity. Depending on these findings, the prepared composite cements were proposed to be used as bone cement and grafting with a function of preventing implant-related infections and post-operative complications after surgery.

**Table 3:** Antimicrobial activity of the Ceramic mixed with gentamicin using agar-well diffusion.

	Inhibition zone (mm)				
	*Pseudomonas aeruginosa*	*Klebsiella pneumonia*	*Staphylococcus aureus*	*Candida albicans*	*Mucor racemosus*
βTCP	9.10 ± 0.11	6.10 ± 0.33	7.15 ± 0.4	4.11 ± 0.02	ND
5BG/βTCP	8.30 ± 0.18	7.20 ± 0.2	8.11 ± 0.15	5.33 ± 0.25	2.16 ± 0.08
10BG/βTCP	9.24 ± 0.45	7.10 ± 0.52	8.21 ± 0.06	7.14 ± 0.15	4.12 ± 0.15
15BG/βTCP	7.40 ± 0.08	8.12 ± 0.25	7.17 ± 0.22	7.33 ± 0.11	3.10 ± 0.2
20BG/βTCP	7.21 ± 0.25	7.10 ± 0.33	8.10 ± 0.1	8.19 ± 0.33	4.09 ± 0.05
Ciprofloxacin	5.13 ± 0.15	7.15 ± 0.05	6.12 ± 0.22	–	
Cephradine	ND	ND	ND	–	
Cephalexin	ND	ND	ND	–	
Fluconazole	–			4.23 ± 0.11	ND
Amphotericin B	–			6.13 ± 0.11	4.09 ± 0.18

### Cytotoxicity results

**Figure 14:**
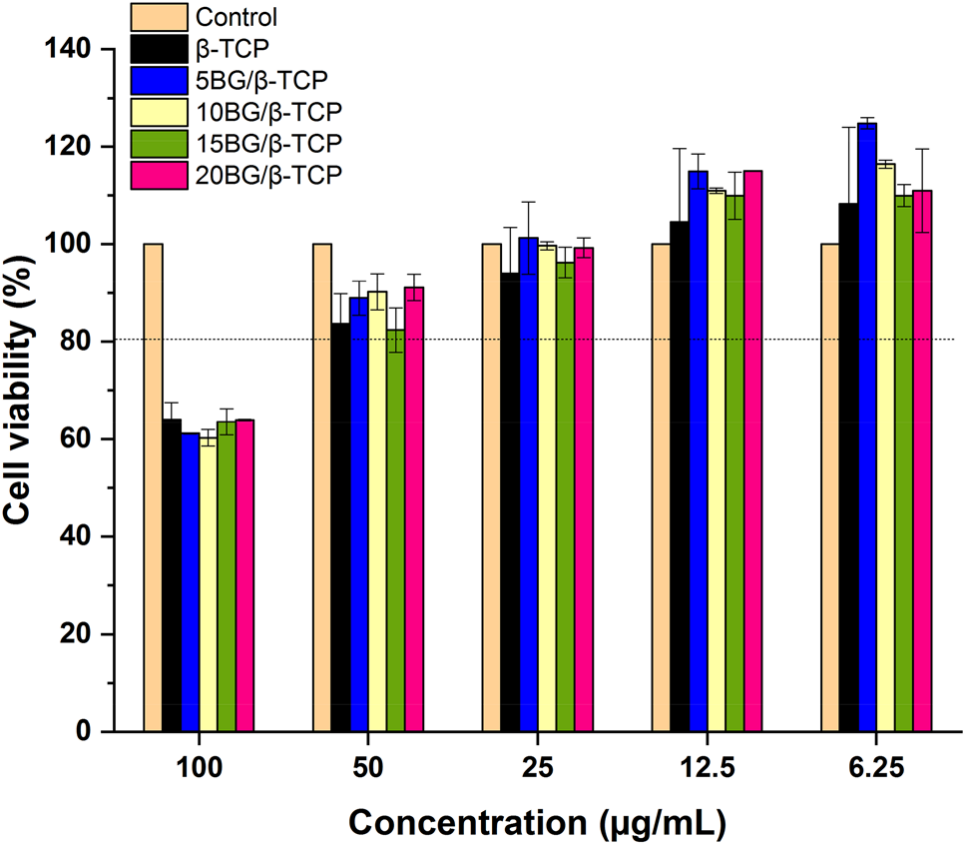
Cell viability % of β-TCP, 5BG/β-TCP, 10BG/β-TCP, 15BG/β-TCP, and 20BG/β-TCP against MG-63 cell line after 48 h of incubation.

Figure 14 shows the viability % of MG-63 cells against different concentrations of the composite cements (6.25–100 µg/mL) using the MTT assay after 48 h incubation. It can be observed from the figure that > 80% of cells were viable with sample concentration up to 50 µg/mL with no more than 7–12% dead cells. Therefore, all samples have weak cytotoxic effects. By extrapolating the dose–response curve (not shown here), the IC_50_ for each sample was calculated to be 178.72, 138.15, 128.8, 186.12, and 137.61 µg/mL for samples β-TCP, 5BG/β-TCP, 10BG/β-TCP, 15BG/β-TCP and 20BG/β-TCP, respectively. So, it can be concluded that all samples were safe on MG-63 cells.

## Conclusion

Novel composites of 5 to 20 weight percent of bioactive glass (BG) added to β-tricalcium phosphate (β-TCP) were created and evaluated. The results demonstrated that 20BG/β-TCP has superior mechanical characteristics and a higher level of bioactivity behavior as a result of the development and growth of a bioactive HA layer on the composite's surface following soaking in SBF solution. XRD, FTIR, and SEM–EDS studies have been used to track the evolution and expansion of the HA layer on the composite surface. The in vitro drug release rate was drastically decreased by addition of BG. Moreover, homogenous combination between the composite cements and gentamicin was proved via the greatest inhibition zone around each concentration of ceramic, since each concentration of cement become valuable toward *Pseudomonas aeruginosa*, *Klebsiella pneumonia*, and *Staphylococcus aureus.* In addition, fungal strains was found to be more affected 10BG/βTCP and 20BG/βTCP samples (i.e. at high percentage of BG) emphasized an excellent homogeneity between them in these concentration against multicellular fungi like *Mucor racemosus.* Cytotoxicity results proved that all samples are safe on MG-63 cells up to 50 µg/mL with no more than 7–12% dead cells. So the prepared composite cements were recommended to be used as bone cement and grafting with a function of preventing implant-related infections and post-operative complications after surgery.

## Data Availability

The datasets generated and/or analyzed during the current study are not publicly available because they are private, but are available from the corresponding author on reasonable request.
